# Fueling Open Innovation for Malaria Transmission-Blocking Drugs: Hundreds of Molecules Targeting Early Parasite Mosquito Stages

**DOI:** 10.3389/fmicb.2019.02134

**Published:** 2019-09-13

**Authors:** Michael Delves, M. Jose Lafuente-Monasterio, Leanna Upton, Andrea Ruecker, Didier Leroy, Francisco-Javier Gamo, Robert Sinden

**Affiliations:** ^1^Department of Infection Biology, Faculty of Infectious and Tropical Diseases, London School of Hygiene & Tropical Medicine, London, United Kingdom; ^2^Department of Life Sciences, Imperial College London, London, United Kingdom; ^3^Diseases of the Developing World (DDW), GlaxoSmithKline, Tres Cantos, Spain; ^4^Mahidol Oxford Tropical Medicine Research Unit, Faculty of Tropical Medicine, Mahidol University, Bangkok, Thailand; ^5^Centre for Tropical Medicine and Global Health, Nuffield Department of Medicine, University of Oxford, Oxford, United Kingdom; ^6^Medicines for Malaria Venture, Geneva, Switzerland

**Keywords:** malaria, transmission, ookinete, drug, screening

## Abstract

**Background:**

Despite recent successes at controlling malaria, progress has stalled with an estimated 219 million cases and 435,000 deaths in 2017 alone. Combined with emerging resistance to front line antimalarial therapies in Southeast Asia, there is an urgent need for new treatment options and novel approaches to halt the spread of malaria. *Plasmodium*, the parasite responsible for malaria propagates through mosquito transmission. This imposes an acute bottleneck on the parasite population and transmission-blocking interventions exploiting this vulnerability are recognized as vital for malaria elimination.

**Methods:**

13,533 small molecules with known activity against *Plasmodium falciparum* asexual parasites were screened for additional transmission-blocking activity in an *ex vivo Plasmodium berghei* ookinete development assay. Active molecules were then counterscreened in dose response against HepG2 cells to determine their activity/cytotoxicity window and selected non-toxic representative molecules were fully profiled in a range of transmission and mosquito infection assays. Furthermore, the entire dataset was compared to other published screens of the same molecules against *P. falciparum* gametocytes and female gametogenesis.

**Results:**

437 molecules inhibited *P. berghei* ookinete formation with an IC_50_ < 10 μM. of which 273 showed >10-fold parasite selectivity compared to activity against HepG2 cells. Active molecules grouped into 49 chemical clusters of three or more molecules, with 25 doublets and 94 singletons. Six molecules representing six major chemical scaffolds confirmed their transmission-blocking activity against *P. falciparum* male and female gametocytes and inhibited *P. berghei* oocyst formation in the standard membrane feeding assay at 1 μM. When screening data in the *P. berghei* development ookinete assay was compared to published screens of the same library in assays against *P. falciparum* gametocytes and female gametogenesis, it was established that each assay identified distinct, but partially overlapping subsets of transmission-blocking molecules. However, selected molecules unique to each assay show transmission-blocking activity in mosquito transmission assays.

**Conclusion:**

The *P. berghei* ookinete development assay is an excellent high throughput assay for efficiently identifying antimalarial molecules targeting early mosquito stage parasite development. Currently no high throughput transmission-blocking assay is capable of identifying all transmission-blocking molecules.

## Introduction

Malaria is still a disease of devastating medical and economic impact affecting nearly half of the world’s population. **Plasmodium**, the apicomplexan parasite responsible for malaria has a complex life cycle requiring both vertebrate hosts and mosquitoes. With every round of asexual replication within the blood, a proportion of cells are triggered to undergo an alternative developmental pathway and transform into mosquito-transmissible male and female gametocytes. When a mosquito bites, these gametocytes are taken up in the bloodmeal and within minutes transform into male and female gametes in the mosquito midgut. Fertilization ensues and the resultant zygote develops into a motile ookinete around 22 h after feeding. The ookinete migrates to and through the midgut wall forming an oocyst and infecting the mosquito. Parasites then further develop into sporozoites that migrate to the salivary glands ready to infect a new human host.

With the exception of tafenoquine and 8-aminoquinolines, all antimalarial drugs have been developed primarily to target the pathogenic asexual stage of the parasite life cycle. Consequently, they have variable activity against other parasite stages possessing divergent cell biology (Delves M. et al., 2012; Plouffe et al., 2016). In order to achieve local elimination and global eradication of malaria, reducing parasite transmission is essential (Rabinovich et al., 2017). At the point of seeking treatment, malarial patients frequently already possess mosquito-infectious male and female gametocyte stage parasites, therefore a patient can be treated and “cured” of their infection but still be able to pass parasites on to mosquitoes and perpetuate the disease (Eziefula et al., 2013). In addition, a significant proportion of the population in malaria-endemic areas have asymptomatic infections and harbor submicroscopic levels of gametocytes which contribute to the persistence of malaria. As gametocytes develop and reside within the human host, gametocyte-targeted transmission-blocking drugs can be administered directly to the patient (Burrows et al., 2017). However, the reduced and divergent metabolism of the gametocyte leaves them insensitive to most antimalarial treatments (Delves M. et al., 2012; Plouffe et al., 2016). Given that the mosquito bloodmeal is composed primarily of blood, transmission-blocking drugs administered to the patient can also access and target early mosquito stage parasite development when the parasite “reawakens” its metabolism and displays more targetable cell biology. However, such a strategy requires drugs with long half-lives so that efficacious concentrations can be maintained within every mosquito bite where infectious gametocytes are present. Alternatively, recent data has shown surfaces treated with the transmission-blocking antimalarial atovaquone are effective at delivering an efficacious dose directly to the mosquito through contact exposure (Paton et al., 2019).

In 2010, GlaxoSmithKline (GSK) reported and released the chemical structures of 13,533 molecules from within their compound library with activity against *P. falciparum* asexual development (Gamo et al., 2010). Since then, the Tres Cantos Antimalarial Set (TCAMS) has been extensively screened in a range of high throughput assays against different parasite stages (Almela et al., 2015; Raphemot et al., 2015; Miguel-Blanco et al., 2017) and yielded new antimalarial candidate molecules (Sanz et al., 2011; Calderón et al., 2012; Williamson et al., 2016). To date, little is known about their activity on early parasite development in the mosquito. Using the rodent malaria model *P. berghei* and an established *ex vivo* ookinete development assay (Pb ODA) which simulates the first 22 h of parasite development in the mosquito (Delves M.J. et al., 2012), we report the screening, identification and profiling of new transmission-blocking molecules.

## Results

### Screening of the TCAMS Library in the Pb ODA

The Pb ODA introduces gametocyte-infected mouse blood to compound-treated “ookinete medium” that simultaneously stimulates gametogenesis *ex vivo*. The readout of this assay is parasite expression of GFP under the control of an ookinete-specific promoter (CTRP) which is detected in a fluorescence plate reader. Therefore, this assay can measure the ability of a compound to inhibit parasite development from the onset of gametogenesis to ookinete formation – approximately the first 22 h of mosquito stage development.

**FIGURE 1:**
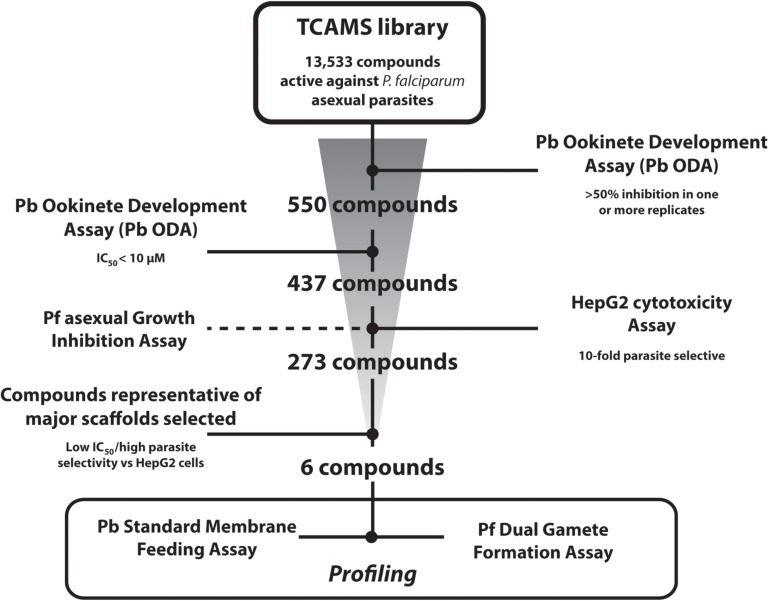
Screening Progression Cascade. The Tres Cantos Antimalarial Set (TCAMS) was screened in the *P. berghei* ookinete development assay (Pb ODA) at 1 μM in duplicate identifying 550 compounds showing >50% inhibition. 437 compounds were reconfirmed in dose response to have an IC_50_ < 10 μM. Active compounds were counterscreened against *P. falciparum* asexuals and HepG2 cells. Six potent and parasite-selective compounds representing major chemical scaffolds were selected and studied in detail.

The entire TCAMS library was screened in the Pb ODA at 1 μM in duplicate independent experiments (Figure 1). A relatively lenient cut-off of >50% inhibition in one or both replicates was selected as the assay “primary-hit” criterion to permit even weakly active compounds to be investigated. Under these conditions, 550 compounds were identified (Supplementary Table S1). 513 of these compounds were available for resupply and were retested in dose response in at least triplicate independent experiments which confirmed an IC_50_ < 10 μM for 437 of them. Compounds with >70% inhibition in the primary screen exhibited a false positive discovery rate of 4.96% (Supplementary Tables S1, S2). Compounds with 50–69% inhibition in the primary screen, however, showed a false positive discovery rate of 31.66%, suggesting future Pb ODA screens should adopt a more stringent hit criteria of >70% inhibition. One compound with a pyridone scaffold (TCMDC-135461) exhibited a sub-nanomolar IC_50_ of < 0.2 nM (the lowest concentration tested in the dose response analysis). 21 additional compounds exhibited low-nanomolar IC_50_s < 100 nM and a further 162 compounds possessed IC_50_s < 1 μM (Supplementary Table S2).

### Initial Profiling

**FIGURE 2:**
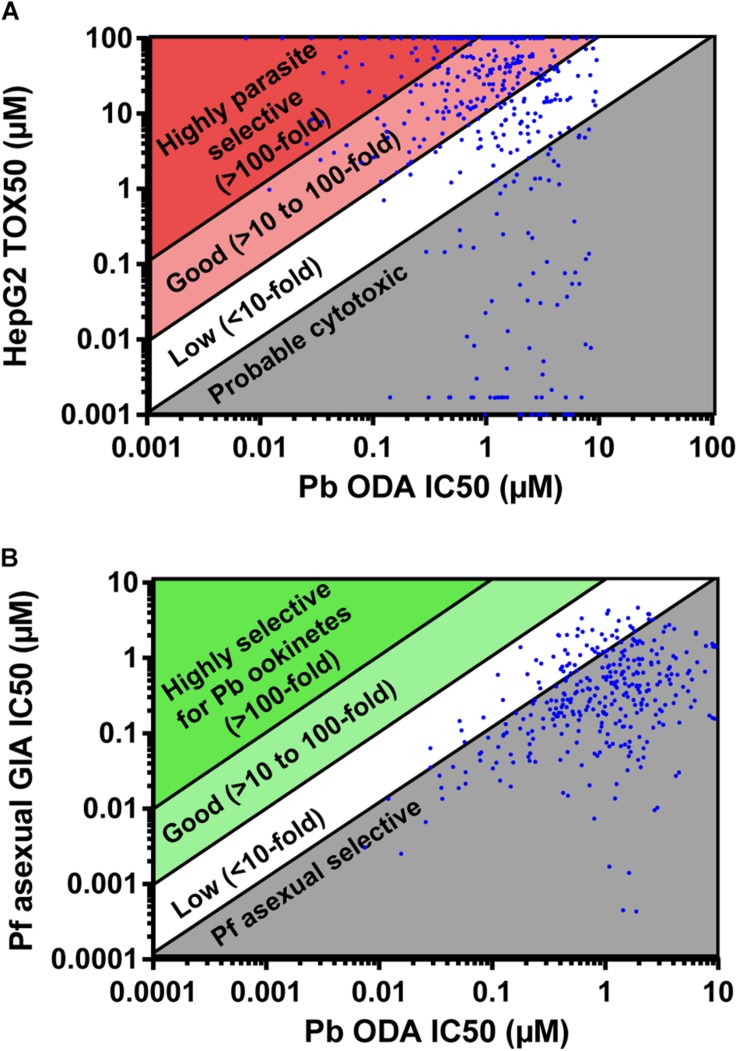
Parasite selectivity and stage specificity. IC_50_s of active compounds were determined in the *P. berghei* ookinete development assay (Pb ODA). **(A)** These were then compared to Tox_50_ values against HepG2 cells to determine parasite selectivity. **(B)** In parallel, to determine parasite stage specificity, Pb ODA IC_50_s were compared to corresponding activity in the *P. falciparum* asexual inhibition growth assay (Pf GIA).

The molecules active against *P. berghei* ookinete development were counter-screened for activity against *P. falciparum* asexual development in the growth inhibition assay (GIA), and additionally screened against HepG2 cells to determine cytotoxicity (Supplementary Table S2). 92 compounds were more potent against HepG2 cells than against ookinete development and therefore were inferred to be cytotoxic (Figure 2A). 77 compounds were slightly parasite selective (1 to 10-fold more active against ookinete development), 190 showed good selectivity (>10 to 100-fold) and 83 were highly selective (>100-fold). After eliminating the cytotoxic compounds, the activity of those remaining were compared to activity in the Pf GIA (Figure 2B). Not a single compound had greater than 8-fold selectivity for *P. berghei* ookinetes over *P. falciparum* asexuals. Given that the TCAMS library is comprised entirely of compounds shown to have activity against *P. falciparum* asexuals, this is unsurprising; indeed 320 out of 441 compounds were more active against asexuals than ookinete development (Figure 2B).

### Chemical Clustering Analysis

**FIGURE 3:**
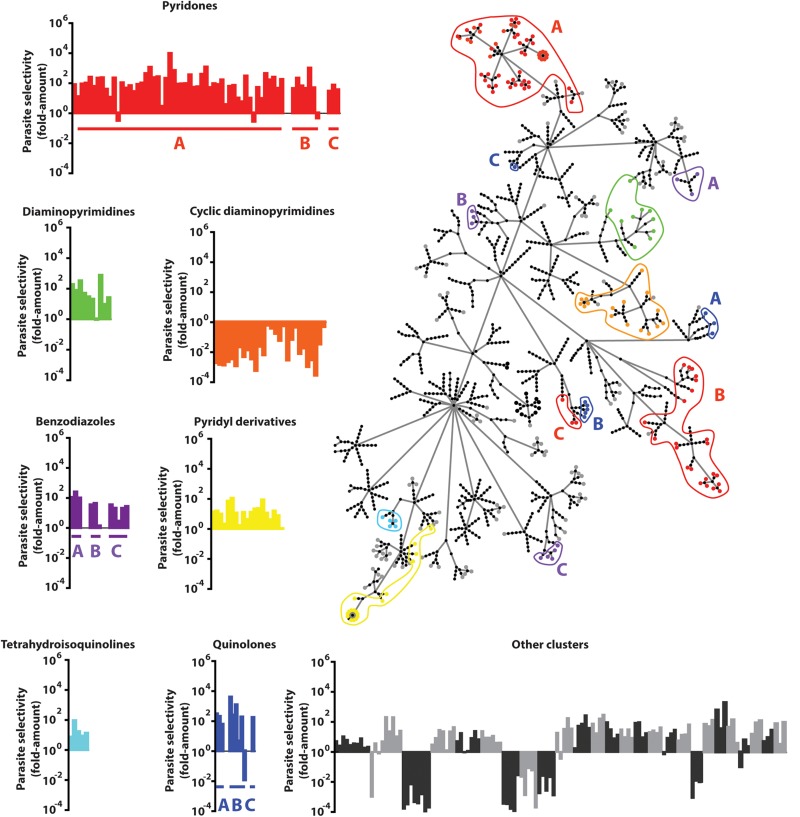
Compound Clustering. Compounds active in the *P. berghei* ookinete development assay (Pb ODA) were clustered using the FragFP algorithm and visualized as a dendrogram. Terminal nodes represent individual compounds and linking nodes represent FragFP similarity toward the single central point in 0.1 unit increments. Colored regions highlight compounds possessing common major scaffolds. Graphs indicate the individual parasite selectivity (HepG2 IC_50_/Pb ODA IC_50_) of members of each scaffold.

Molecular frameworks and fingerprint cluster analyses were used to structurally characterize the 437 confirmed hits, identifying 49 clusters and 128 singletons (Figure 3 and Supplementary Table S2). In addition, many clusters contained compounds that shared a common functional scaffold and were grouped into seven different families comprising of pyridones, cyclic diaminopyrimidines, pyridyl derivatives, diaminopyrimidines, quinolones, tetrahydroisoquinolines, and benzodiazoles. The majority of compounds in the cyclic diaminopyrimidine group were found to be cytotoxic, however, most members of the other groups showed >10-fold parasite selectivity.

### Profiling the Transmission-Blocking Properties of Selected Molecules

**FIGURE 4:**
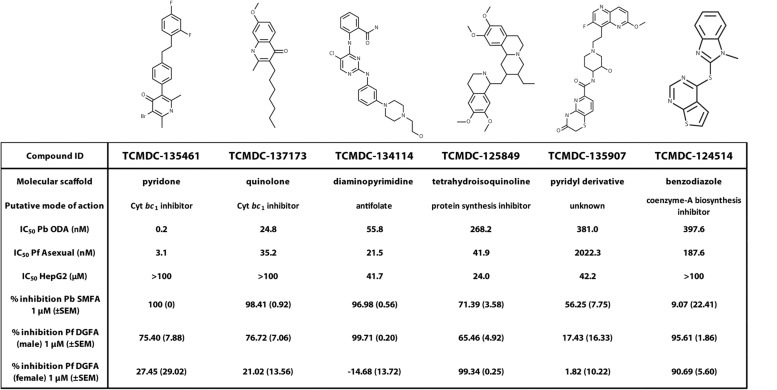
Parasitological profiling. Six compounds representative of each of the major chemical scaffolds (pyridones, cyclic diaminopyrimidines, pyridyl derivatives, diaminopyrimidines, quinolones, tetrahydroisoquinolines and benzodiazoles) with potent activity against ookinetes and high parasite selectivity were selected for profiling in the *P. berghei* standard membrane feeding assay (Pb SMFA) and *P. falciparum* Dual Gamete Formation Assay (Pf DGFA).

Exemplar molecules from the remaining six families showing high potency and parasite selectivity were selected for detailed parasitological profiling: TCMDC-135461 (pyridone); TCMDC-135907 (pyridyl derivative); TCMDC-134114 (diaminopyrimidine); TCMDC-137173 (quinolone); TCMDC-125849 (tetrahydroisoquinoline); TCMDC-124514 (benzodiazole) (Figure 4).

**FIGURE 5:**
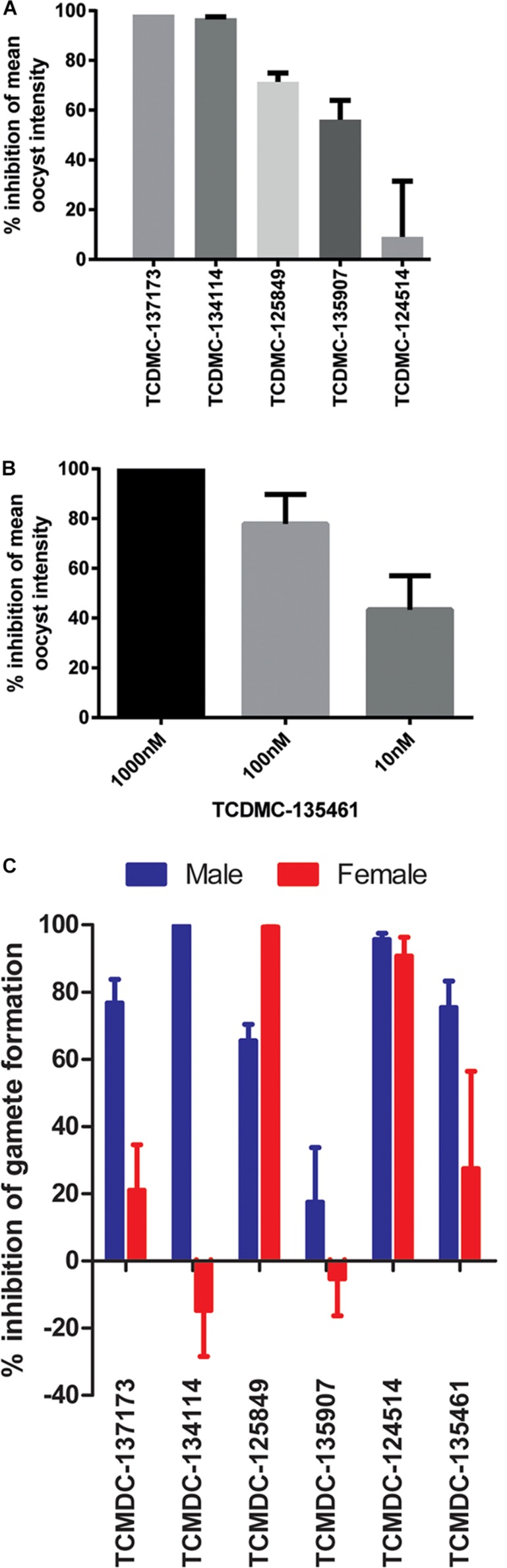
Activity in other transmission-blocking assays. The six selected molecules were evaluated in the *P. berghei* standard membrane feeding assay (Pb SMFA). **(A)** Five molecules were tested at 1 μM to confirm transmission-blocking activity (*n* = 48–53 mosquitoes per treatment). Bars represent the mean of three biological repeats, error bars indicate the standard error of the mean (SEM) **(B)** The most potent compound in the Pb ODA, TCDMC-135461, was investigated over a range of concentrations in Pb SMFAs (*n* = 22–83 mosquitoes per condition). Bars represent the mean of three biological repeats, error bars indicate the standard error of the mean (SEM). **(C)** Additionally, the compounds were tested against male and female *P. falciparum* gametocytes at 1 μM in the Pf DGFA. Bars represent the mean of at least three biological repeats, error bars indicate the standard error of the mean (SEM).

The selected molecules were evaluated for their ability to prevent mosquito transmission at 1 μM in *P. berghei* standard membrane feeding assays (Pb SMFA) (Figure 5A and Supplementary Table S3). Relative activity in the Pb SMFA broadly matched their activity in the Pb ODA, albeit with reduced potency. TCMDC-137173 and TCMDC-134114 both displayed low nanomolar activity in the Pb ODA (IC_50_s < 60 nM). This translated into a 97–100% inhibition of oocyst intensity at 1 μM in the Pb SMFA. TCMDC-125849, TCMDC-135907 and TCMDC-124514 were less active in the Pb ODA (IC_50_s > 250 nM) which translated into a 9–71% inhibition of oocyst intensity at 1 μM in the Pb SMFA. TCMDC-135461, the pyridone compound with the most potent activity in the Pb ODA was tested in the Pb SMFA in dose response at 1 μM, 100 nM, and 10 nM (Figure 5B and Supplementary Table S4). At 1 μM, no oocysts were observed in any mosquitoes. At 100 nM, oocyst intensity was reduced by 77.9% (±11.8% standard error of the mean − SEM). At 10 nM, oocyst intensity was inhibited by 43% (±13.7% SEM). Taken together, based upon the direct comparison of these six compounds in the Pb ODA and Pb SMFA, it would appear that a Pb ODA IC_50_ of at least ∼60 nM translates into a near-total transmission-blockade in mosquito feeds at 1 μM and should set the approximate threshold onward progression of molecules in future screens.

To ensure that data obtained using a rodent malaria parasite shows applicability to human malaria-infective species, the six molecules were tested in an established *P. falciparum* Dual Gamete Formation Assay (Pf DGFA). The Pf DGFA evaluates the ability of test molecules to prevent male and female gametocytes from differentiating into gametes *in vitro*, which is the first step of parasite development in the mosquito (Ruecker et al., 2014; Delves et al., 2018). Activity in the Pf DGFA has been shown to be highly predictive of activity in *P. falciparum* SMFAs which are technically difficult and expensive to perform (Ruecker et al., 2014; Baragaña et al., 2015). At 1 μM a diverse range of activities was observed against male and female *P. falciparum* gametocytes (Figure 5C and Supplementary Table S4). TCMDC-135461 and TCMDC-137173 – the two most potent compounds in the Pb ODA showed 75.4 and 76.7% inhibition of male gametocyte functional viability respectively, but low activity against female gametocytes (27.4 and 21.0% inhibition respectively). This indicates that these compounds are more active against male gametocytes than female gametocytes. TCMDC-134114 gave 97.0% inhibition of male gametocyte functional viability and was not active against female gametocytes – suggesting a male-specific mode of action. In contrast, TCMDC-125849 and TCMDC-124514 showed activity against both male (65.5 and 95.6% inhibition respectively) and female (99.3 and 90.7% inhibition respectively) gametocytes suggesting a mode of action targeting biochemical pathways fundamental to both gametocyte sexes. Finally, TCMDC-135907 showed little-to-no activity in the Pf DGFA, perhaps suggesting that this compound targets parasite development post-gamete formation (hence active in the Pb ODA and not Pf DGFA).

### Comparison to Other Transmission-Blocking Assays

**FIGURE 6:**
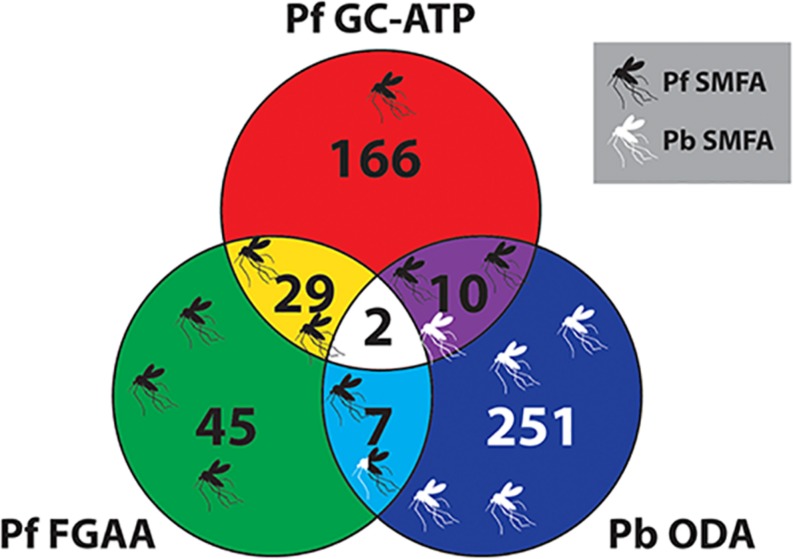
Venn diagram comparing the TCAMS transmission-blocking across three transmission-blocking assay. Active compounds from the *P. berghei* ookinete development assay (Pb ODA) were compared with published activity against *P. falciparum* late stage gametocytes and *P. falciparum* female gametogenesis (Almela et al., 2015; Miguel-Blanco et al., 2017). To ensure parasite specificity, to be classed as active in a particular assay, parasite selectivity had to be greater than ten-fold compared to HepG2 cells (Supplementary Table S5). Where specific compounds have been reported to block transmission in mosquito feeds, this has been mapped onto the Venn diagram as mosquitoes. Their position on the diagram indicates their activity profile in the three assays and the color represents Pf SMFA (Black), Pb SMFA (White) or both (Black and White).

With regards to potential for transmission-blocking, the TCAMS library has also been previously screened against purified *P. falciparum* late stage (stages IV-V) gametocytes in an ATP depletion assay (Pf GC-ATP) (Almela et al., 2015), and purified *P. falciparum* female gametocytes in a female gametogenesis assay (Pf FGAA) (Miguel-Blanco et al., 2017). In the Pf GC-ATP assay, the TCAMS were initially screened at 5 μM and yielded 363 molecules with an IC_50_ < 10 μM, and in the Pf FGAA they were screened at 2 μM, yielding 405 active compounds. In parallel, both studies also performed counterscreens against HepG2 cells for cytotoxicity. Combining these data with the Pb ODA screen results reported here and only considering molecules with a >10-fold parasite specificity in their respective assay gives a collection of transmission-blocking molecules with a range of different phenotypes (Figure 6 and Supplementary Table S5). Nevertheless, molecules from all three assays have demonstrated transmission-blocking activity in mosquito feeds (Almela et al., 2015; Miguel-Blanco et al., 2017; Colmenarejo et al., 2018).

## Discussion

The TCAMS library is the largest collection of antimalarial molecules in the public domain and has sparked several major antimalarial drug discovery campaigns. Antimalarial molecules that have additional transmission-blocking activity have the potential to make a significant contribution to malaria elimination and eradication and are prioritized for development (Burrows et al., 2017). The Pb ODA is a simple and relatively inexpensive assay that can characterize the transmission-blocking activity of hundreds of molecules with the gametocyte infected blood of a single mouse (compared to Pb SMFAs which can evaluate ∼2 molecules per mouse). It correlates well with SMFA data and the majority of identified molecules are also active against *P. falciparum* sexual stages.

TCMDC-135461, the representative pyridone molecule selected for profiling was the most potent molecule active in the Pb ODA but was less active against male and female gametocytes in the Pf DGFA. Other pyridones are known to act by targeting the parasite respiratory chain by inhibiting cytochrome *bc*_1_ (Capper et al., 2015). Supporting this activity, mitochondrial respiration is essential for mosquito stage parasite development (Goodman et al., 2016) and other cytochrome *bc*_1_ inhibitors such as atovaquone are established transmission-blocking molecules (Fowler et al., 1995). However, phase-1 clinical trials of the pyridone antimalarial molecule GSK932121 were halted after it showed acute cardiotoxicity in rats, thought to be through inhibition of mammalian cytochrome *bc*_1_ (Capper et al., 2015). This suggests higher selectivity is required for other pyridones to be used as orally administered antimalarials. Recently, contact exposure of mosquitoes to atovaquone has shown to inhibit parasite development in the mosquito (Paton et al., 2019). This reinforces the potential of parasite cytochrome *bc*_1_ inhibitors as transmission blocking agents. Given the extreme potency of TCMDC-135461, indoor residual spraying and/or baited sugar traps may be a practical, efficacious and safer method of utilizing this class of molecules to elicit a transmission-blocking response. TCMDC-134114, the representative diaminopyrimidine molecule was also potent in the Pb ODA and Pb SMFA. Interestingly it had male gametocyte-specific activity in the Pf DGFA. The diaminopyrimidine scaffold is found in many molecules targeting dihydrofolate reductase (DHFR), such as the antimalarial pyrimethamine. DHFR is required for parasite folate biosynthesis which is essential for generating the nucleotides required for DNA replication (Heinberg and Kirkman, 2015). Male gametogenesis requires three rounds of endomitosis to generate eight male gametes. In addition, ookinete development requires one round of meiosis. Therefore, activity in the Pb ODA (which includes male gametogenesis and ookinete development) and male-specific activity against gametocytes in the Pf DGFA is consistent with this mode of action for TCMDC-134114. Datamining the chemical structure of TCMDC-137173 (the representative quinolone compound) revealed that it is the established antimalarial quinolone endochin which was first reported in 1948 (Doggett et al., 2012). Since then, several endochin-like antimalarials have been developed, however, poor oral bioavailability has hampered their clinical progress (Nilsen et al., 2013; Miley et al., 2015). Like the pyridones, TCMDC-137173/endochin targets the parasite mitochondrial cytochrome *bc*_1_ complex and therefore displays a transmission-blocking profile similar to TCMDC-135461. TCMDC-125849, the representative tetrahydroisoquinoline was moderately active in the Pb ODA with an IC_50_ of 268 nM. Reflecting this, it gave only 71.4% inhibition of oocyst intensity in the Pb SMFA at 1 μM. Supporting this level of transmission-blocking activity, 2 μM TCMDC-125849 reportedly gives 100% inhibition of oocyst intensity and total inhibition of male gametogenesis in *P. falciparum* (Colmenarejo et al., 2018). The chemical structure of TCMDC-125849 closely resembles emetine – a known antiprotozoal compound which inhibits protein synthesis (Toyé et al., 1977; Wong et al., 2014). Supporting this hypothesis of targeting such a fundamental biological pathway, 1 μM TCMDC-125849 inhibited both male and female gametogenesis (Figure 5C). In particular, female gametogenesis was inhibited by 99.3%. The female readout of the Pf DGFA relies on antibody detection of female gamete expression of Pfs25, which is held under translational repression in the female gametocyte. Therefore, inhibition of protein synthesis by TCMDC-125849 would be predicted to strongly reduce Pfs25 expression as observed. The representative benzodiazole TCMDC-124514 was also moderately active in the Pb ODA with an IC_50_ of 397 nM which was reflected by low inhibition of oocyst intensity (9.1% inhibition) in the Pb SMFA. Interestingly at 1 μM it potently inhibited both male and female gametogenesis suggesting it targets biology fundamental to both gametocyte sexes. In support of this, TCMDC-124514 has been hypothesized to be a Coenzyme A biosynthesis inhibitor (Weidner et al., 2017) which would both affect energy and lipid metabolism – both fundamental pathways within the parasite. The difference in species activity suggest that TCMDC-124514 is more active against *P. falciparum* than *P. berghei*. The representative pyridyl derivative, TCMDC-135907 showed moderate activity in the Pb ODA with an IC_50_ of 381 nM, which again was reflected incomplete inhibition at 1 μM in the Pb SMFA. There is no data in the literature to suggest a mode of action for this compound, however, data presented here suggests it targets the parasite after gamete formation.

When comparing the activities of several high throughput transmission assays against the TCAMS collection, it is clear to see that no single assay is sufficient to identify all transmission-blocking molecules. Likely this is due to the distinct (but overlapping) cell biology that they interrogate. To date, a high throughput assay that incorporates the entire cell biology of transmission does not exist. Therefore, to maximize the discovery of new transmission-blocking antimalarials, it would be prudent to use several assays and not rely on the data generated by a single assay alone.

## Materials and Methods

### Compound Handling

The TCAMS library was provided in duplicate on assay-ready 384 well plates by GSK. 50 nl of each compound in 100% DMSO was stamped into each well with one column containing DMSO-only (negative control) and one column containing cycloheximide at a final assay concentration of 10 μM (positive control). Assay plates were stored at −20°C until used. Compounds selected for further investigation were supplied by GSK as 10 mM stock solutions in 100% DMSO.

### *P. berghei* Ookinete Development Assay (Pb ODA)

All work involving laboratory animals was performed in accordance with the EU regulations EU Directive 86/609/EEC and within the regulations of the United Kingdom Animals (Scientific Procedures) Act 1986. The Pb ODA was performed exactly as described in Delves M.J. et al. (2012). Female T0 mice were treated with an intraperitoneal (ip) injection of 200 μl 6 mg/ml phenylhydrazine to induce hyperreticulocytosis. Three days later, the treated mice were infected with approximately 10^8^ parasites by ip injection of blood from a donor mouse infected with *Plasmodium berghei* expressing GFP under the control of the ookinete-specific CTRP promoter (Vlachou et al., 2004). Three days afterward, the presence of high levels of gametocytes were confirmed by stimulating and observing male gametogenesis (exflagellation). A drop of blood was sampled from the tail of each mouse and mixed with ookinete medium (RPMI medium with 25 mM HEPES, 2 mM L-glutamine, 2 g/l sodium bicarbonate, 50 mg/l hypoxanthine and 100 μM anthurenic acid, adjusted to pH 7.4, plus 20% FBS) under a coverslip on a glass slide. Ten minutes later vigorously beating exflagellation centers were observed by phase contrast microscopy and invariably showed >10 centers per field at ×40 objective. Infected mice were anesthetized in batches of five, rapidly exsanguinated, blood pooled and immediately transferred to ookinete medium at a 1:20 dilution. 50 μl of blood/medium was then rapidly dispensed into each well of the assay plates using a Multidrop Combi automated dispenser (Thermo Scientific) and the plates incubated in a humidified incubator at 19°C in the dark for 22 h. GFP fluorescence was then measured in a fluorescence plate reader (BMG Labtech FluoSTAR Omega) and inhibition of ookinete production calculated in relation to the positive and negative controls using the formula:% inhibition of ookinete production = 100 − [(well_fluorescence − positive_control)/(negative_control − positive_control) × 100].

### Compound Clustering and Computational Analysis

Clustering was performed following the computational methods described in Gamo et al. (2010). Briefly, molecular frameworks were calculated using an in-house implementation of the algorithm described previously (Bemis and Murcko, 1996). Compounds were also classified in chemical families using the Daylight fingerprint methods with a Tanimoto similarity index of 0.85, following the procedures in the Daylight Information Systems manual (Daylight Chemical Information Systems, 2019). The dendrogram in Figure 3 was constructed using data obtained from the FragFP clustering algorithm performed within OSIRIS DataWarrior Version 04.07.03 and visualized using Cytoscape Version 3.7.1.

### *P. berghei* Standard Membrane Feeding Assays (Pb SMFAs)

Membrane feeding assays were performed essentially as described in Ramakrishnan et al. (2013). Briefly, a phenylhydrazine-treated mouse (see above) was infected with blood from a donor mouse infected with *P. berghei* 507 parasites that constitutively express GFP throughout the parasite life cycle (Janse et al., 2006). Three days later the infected mouse was rapidly exsanguinated and the parasite-infected blood diluted at 1:10 dilution in naïve mouse blood pre-warmed to 37°C. Working as quickly as possible, the blood mix was divided into 500 μl aliquots and mixed with test compounds or DMSO alone at appropriate concentrations (final DMSO concentration of 0.2%) and immediately added to plastic water-jacketed membrane feeders and fed to *Anopheles stephensi* SDA 500 strain mosquitoes. After the feed, mosquitoes were maintained at 19°C/80% relative humidity overnight before unfed mosquitoes were removed. Fed mosquitoes continued to be maintained on 8% fructose (w/v)/0.05% (w/v) p-aminobenzoic acid for 7 days before their midguts were dissected out and oocyst burden quantified using fluorescence microscopy and semi-automated counting analysis (Delves and Sinden, 2010). Data presented is the mean of three independent experiments.

### *P. falciparum* Dual Gamete Formation Assay (Pf DGFA)

Compounds were tested exactly as described in Ruecker et al. (2014) in the carry-over assay format. Briefly, functionally mature stage V male and female gametocytes were generated by seeding *P. falciparum* NF54 strain asexual cultures at 1% parasitemia and 4% hematocrit and replacing culture medium (RPMI medium with 25 mM HEPES, 2 mM L-glutamine, 2 g/l sodium bicarbonate, 50 mg/l hypoxanthine plus 10% human serum) daily for 14 days whilst maintaining a constant temperature of 37°C and a gas mix of 3% CO_2_, 5%O_2_, 92% N_2_. Gametocyte cultures were diluted to 25 million cells/ml (including erythrocytes) and 200 μl added to each well of a round-bottomed 96 well plate containing test compounds. Plates also contained DMSO negative and 20 μM methylene blue positive control wells. After a 24 h incubation at 37°C, gametogenesis was stimulated by transferring the gametocytes to a flat-bottomed 96 well plate containing 10 μl of ookinete medium at room temperature. Twenty minutes later, the emergence of male gametes was recorded microscopically using phase contrast and ×10 objective lens. The plate was then further incubated at 26°C for another 24 h to allow emerged female gametes to maximally express Pfs25 on their cell surface. An anti-Pfs25 antibody (Mab 4B7) conjugated to the Cy3 fluorophore was added to the plates and female gametes detected by live fluorescence microscopy at ×10 objective. Data was then processed using custom image analysis algorithms and percent inhibition in respect to positive (methylene blue) and negative (DMSO) controls was calculated. Data presented is the mean of four independent biological repeats.

### *P. falciparum* Asexual Growth Inhibition Assay (Pf GIA)

The asexual activity of active compounds was tested in dose response with parasite lactate dehydrogenase (LDH) activity used as a readout of parasite viability as described in Gamo et al. (2010). Briefly, 3D7-strain *P. falciparum* asexual parasites were diluted to 0.25% parasitemia, 2% hematocrit in culture medium composed of RPMI medium with 25 mM HEPES, 2% D-sucrose, 0.3% L-glutamine, 150 μM hypoxanthine plus 5% albumax. 25 μl of parasite inoculum was then dispensed into each well of a 384 well plate containing test compounds and positive (50 μM chloroquine) and negative (DMSO) controls. Plates were incubated for 72 h at 37°C in a 5% CO_2_, 5%O_2_, 90% N_2_ environment before being frozen at −70°C. Plates were then thawed and 70 μl of reaction mix (143 mM sodium L-lactate, 143 mM 3-acetyl pyridine adenine dinucleotide, 178.75 mM Nitro Blue tetrazolium chloride, 286 mg/ml diaphorase (2.83 U/ml), 0.7% Tween 20, 100 mM Tris–HCl pH 8.0) was added to each well using a Multidrop Combi automated dispenser. Plates were shaken to mix and then absorbance at 650 nm was measured after 10 min incubation at room temperature. Percent inhibition of asexual growth was then calculated with respect to positive and negative controls. Data presented is the mean of three independent biological replicates.

### HepG2 Cytotoxicity Assay

The activity of active compounds against HepG2 cells was performed as described in Delves et al. (2018). Briefly, HepG2 cells were seeded onto compound-treated 384 well plates at a density of 2,500 cells per well in Minimal Essential Medium (MEM) plus 10% fetal bovine serum and 1% non-essential amino acids solution (NEAA). Cells were incubated with test compounds or 50 μM doxorubicin (positive) and DSMO (negative) controls at 37°C for 48 h in a humidified 5% CO_2_ atmosphere. Plates were then treated with Resazurin to a final concentration of 45 μM for 4 h. Cell viability was then measured in a fluorescent plate reader and percent inhibition calculated with respect to positive and negative controls.

## Data Availability

All datasets generated for this study are included in the manuscript and/or Supplementary Files.

## Ethics Statement

The animal study was reviewed and approved by Imperial College Animal Welfare Ethical Review Body.

## Author Contributions

MD, RS, F-JG, and DL designed the study. MD performed the Pb ookinete assay. ML-M performed the chemical clustering analysis. MD and LU performed the Pb SMFAs. AR performed the Pf DGFA.

## Conflict of Interest Statement

F-JG and ML-M are employed by GlaxoSmithKline. The remaining authors declare that the research was conducted in the absence of any commercial or financial relationships that could be construed as a potential conflict of interest.
